# MAP3K8 is a potential therapeutic target in airway epithelial inflammation

**DOI:** 10.1186/s12950-024-00400-2

**Published:** 2024-07-19

**Authors:** Chih-Yung Chiu, Saffron A. G. Willis-Owen, Kenny C.C. Wong, Stuart N. Farrow, William O.C. Cookson, Miriam F. Moffatt, Youming Zhang

**Affiliations:** 1 National Heart and Lung Institute, Imperial College London, SW3 6LY London, UK; 2 Division of Paediatric Pulmonology, Chang Gung Memorial Hospital Linkou, Chang Gung University College of Medicine, Taoyuan, Taiwan; 3 Cancer Research Horizons, Babraham campus, Cambridge, CB22 3AT UK

**Keywords:** MAP3K8, Pathways, Epithelial cells, Inflammation

## Abstract

**Background:**

We have previously discovered clusters of sequentially negative and positive modulators of acute inflammation during cytokine stimulation in epithelial cells and identified potential targets for therapy within these clusters. MAP3K8 is a druggable kinase that we found to be a hub of a principal interaction network. We describe here the results of *MAP3K8* knockdown in the A549 lung cancer cell line, the BEAS-2B epithelial cell line and normal human bronchial epithelial (NHBE) cells following IL-1β stimulation. We analysed signalling transduction and global gene expression after IL-1β stimulation with and without *MAP3K8* knockdown, quantifying levels of the inflammatory cytokines IL-6, IL-8 and RANTES levels by qPCRs and/or by ELISAs. We also examined potential small molecule inhibitors for MAP3K8 in the same models.

**Results:**

IL-1β significantly and consistently increased MAP3K8 expression after 2 h in A549, BEAS-2B and NHBE cells. Phosphorylation of MAP3K8 occurred at 20 min after IL-1β stimulation and MAP3K8 protein was degraded at 30 min. *MAP3K8* knockdown significantly reduced IL-6, IL-8 levels after IL-1β stimulation and yielded a 10-fold enhancement of the anti-inflammatory effects of dexamethasone. Phosphorylation of ERK1/2 (P-ERK1/2) and phosphorylation of SAPK/JNK (P-SAPK/JNK) decreased at 30 min after IL-1β stimulation with *MAP3K8* knockdown. The combination of dexamethasone and *MAP3K8* knockdown resulted in greater inhibition of phosphorylated ERK1/2 and SAPK/JNK. Nineteen genes including *MMP1*,* MMP3*,* MMP10*,* ITGB8*,* LAMC2* and *PLAT* (*P* corrected < 0.01 respectively) demonstrated a distinct altered temporal response to IL-1β following suppression of *MAP3K8*. However, putative MAP3K8 inhibitors including Tpl2-1, Tpl2-2 and GSK2222867A only showed inhibition of IL-6 and IL-8 production at a high dose.

**Conclusions:**

These results confirm that MAP3K8 is a key mediator of the early inflammatory response and that it is a potential target in inflammatory diseases. However, current tool compounds do not effectively inhibit its effects.

## Background

Airway inflammation is one of the central characteristics of chronic respiratory diseases such as asthma and chronic obstructive pulmonary disease (COPD). MAPK transduction signalling pathways are major regulatory influence on in the airway inflammatory response. MAPK kinase kinase 8 (MAP3K8) (also known as tumour progression locus 2 (TPL2)) is a cancer Osaka thyroid (COT) oncogene. It was first identified by virtue of its oncogenic transforming activity in cells [1, 2]. It is a mitogen-activated protein kinase kinase (MAP3K) that is activated downstream post engagement of the receptors TNFαR, IL-1R and TLR [3]. When over-expressed, MAP3K8 activates MEK, ERK, JNK, p38 MAPK and ERK5, influencing the growth and survival of cells in combination with other signalling pathways, such as nuclear factor kappa-B (NFKB), tumour necrosis factor (TNF) and interleukin-1 (IL-1) [4, 5].

In our previous experiments, designed to assess the global transcriptional regulation of the inflammatory response in airway epithelium, we identified 336 genes that showed differential expression (≥ log2 fold change and significant at a 1% false discovery rate (FDR)) across an experimental period of 12 h. The transcripts were organised into five strongly and sequentially regulated clusters [6]. *MAP3K8* was the hub gene for the early hours component cluster (peak expression at 2 h) of the inflammatory response (manuscript in preparation).

*MAP3K8* is located on human chromosome 10 and has 9 exons. It is primarily expressed in the immune system, but can also be found in tissue resident stromal cells [7]. The gene encodes two isoforms to produce 52 KDa or 58 KDa proteins respectively [8, 9]. Recent studies have shown that MAP3K8 has important roles in multiple inflammatory diseases including inflammatory bowel disease (IBD) [10], rheumatoid arthritis (RA) [11], multiple sclerosis (MS) [12], intestinal inflammation [13] and lung inflammation [1, 3, 14]; suggesting that MAP3K8 regulates signalling pathways that are shared amongst the inflammatory diseases. Understanding how MAP3K8 influences the inflammatory response, and the following downstream signalling, therefore has the potential to provide targets for a range of common diseases that have a significant inflammatory component to them.

In this report, we established epithelial cell models of inflammation with IL-1β stimulation and systematically analysed the effect of knockdown. We analysed the MAP3K8 signal transduction pathways investigating the impact of steroid on the inflammatory response with and without *MAP3K8* knockdown. We also performed global gene expression analysis in *MAP3K8* knockdown airway epithelial cells and investigated the potential MAP3K8 inhibitors assessing their anti-inflammatory efficacies in the airway.

## Methods

### Airway epithelial cell culture

A549 human lung epithelial cells (American Type Culture Collection (ATCC)) were cultured in Dulbecco’s Modified Eagle’s Medium (DMEM) containing 10% (vol/vol) FCS and 2mM L-Glutamine. Cells were used at passage numbers 90–110. Cells from the transformed human bronchial epithelial cell line BEAS-2B were grown in keratinocyte serum-free medium (K-SFM; GIBCO BRL) supplemented with recombinant human epidermal growth factor (rhEGF) and bovine pituitary extract (PE). BEAS-2B cells were obtained from ATCC and were used at passage numbers 60–80. NHBE cells were obtained from Lonza and cultured to a maximum of five passages to limit variable responses. The NHBE cells were grown in bronchial epithelial medium (BEGM; Lonza) containing a mixture of growth factors, cytokines, and supplements (BulletKit; Lonza). All cells were maintained in 150 cm^2^ (T150) flasks with 5% CO2 at 37 °C. Medium was replaced every second day, and cells were passaged when > 85% confluent by washing with Dulbecco’s Phosphate Buffered Saline (DPBS) and dislodging with 0.5% trypsin. Cell viability was determined microscopically by trypan blue (Sigma-Aldrich) exclusion, and cell numbers counted by haemocytometer.

### Inhibitors, antibodies, and cytokine assays

The putative MAP3K8 kinase inhibitor Tpl2-1 was purchased from Santa Cruz Biotechnology Inc., the inhibitor Tpl2-2 was purchased from Calbiochem. GSK2222284A and GSK2222867A, novel MAP3K8 kinase inhibitors, were gift from GlaxoSmithKline PLC. Anti-MAP3K8 was purchased from Santa Cruz Biotechnology Inc. and phospho-MAP3K8 (T290) was from Invitrogen Corp. Anti-MEK1/2, anti-p-MEK-1/2, anti-SEK1/MKK4, anti-phospho-SEK1/MKK4, anti-MKK7, anti-phospho-MKK7, anti-p44/42 MAPK, anti-phospho-p44/42 MAPK, anti-SAPK/JNK, anti-phospho-SAPK/JNK, anti-p38α MAPK, anti-phospho-p38 MAPK antibodies were purchased from Cell Signaling Technology. Other antibodies and horseradish peroxidase (HRP) used for Western blotting were purchased from DAKO. Human IL-6, IL-8, and RANTES were measured by ELISA kits according to manufacturer’s instructions (DuoSet; R&D Systems Europe).

### Gene knockdown reagents and protocols

RNA interference (RNAi) was carried using ON-TARGETplus SMARTpool (Dharmacon Research Inc.) and ON-TARGETplus. Non-targeting Pool negatives were used as controls. RNAi transfection of A549 cells was performed using a previously established protocol [15] by using Lipofectamine RNAiMAX (Invitrogen Corp.). Briefly cells were seeded into 24-well plates (Corning Costar Corp.) at 4 × 10^4^ cells/well to ensure 40–60% confluence was established by the day of transfection. RNAi- Lipofectamine RNAiMAX complexes were formed by adding 2 µl/ml of Lipofectamine RNAiMAX and 50nM of RNAi in serum free medium (Sigma-Aldrich) followed by incubation for 15 min at room temperature. RNAi-Lipofectamine RNAiMAX complexes were then added to a single well and the cells were placed in a CO2 incubator at 37 °C for 24 h. After this initial transfection, RNAi transfection was repeated with addition of a second dose of RNAi-Lipofectamine RNAiMAX complex followed by further incubation at 37 °C for 24 h. Following double transfection, cells were starved in serum free medium for 16 h and then stimulated with 1 ng/ml of IL-1β (R&D Systems, Europe Ltd., UK). Cells were collected and proteins extracted for Western blotting analysis whilst supernatants were harvested for cytokine measurement.

### Models of IL-1β-induced inflammation

Airway epithelial cells were grown up in T150 flasks (for media details see Airway epithelial cell culture). When greater than 85% confluent, cells were dislodged from the flask surface by addition of trypsin. Wells of a 24-well plates were then seeded with 4 × 10^4^ cells in 1 ml of complete medium/well and incubated overnight in a CO2 incubator at 37^o^C to allow cells attachment and growth. Prior to addition of the IL-1β stimulus, cells were starved in serum-free media for 16 h in order to achieve cell cycle synchronization. After stimulation with 1 ng/ml IL-1β, cells were harvested for *MAP3K8* gene expression at 2, 4, 6, and 8 h, and harvested for MAP3K8 protein measurement at 5, 10, 20, 30, 45 and 60 min. For the *MAP3K8* siRNA knockdown experiments, MAP3K8 protein and phosphorylation of the associated MAPK signalling proteins were measured at 20 and 30 min, whilst inflammatory cytokine gene expression (IL-6 and IL-8) was determined at 2, 4, and 6 h, with cytokine protein release measured at 8 and 24 h. All timings are post IL-1β stimulation.

### Real-time quantitative PCR

Total RNA was isolated from A549 cells using the Qiagen RNeasy mini kit following the manufacturer’s instructions. Reverse transcription was performed on 0.5 µg of RNA and cDNA was synthesized using the High-Capacity cDNA Reverse Transcription Kits (Applied Biosystems) following manufacturer’s instructions. Real-time PCR was performed using the Rotor-Gene 3000™ Real-Time PCR detection system (Corbett Research) with the Platinum SYBR Green qPCR SuperMix-UDG (Invitrogen). Data were normalized by the level of GAPDH expression in individual samples. Calculation of the threshold value, standard curve preparation and quantification of mRNA in the samples were performed using the Rotor-Gene 6.0 software (Corbett Research).

### Western blotting

Whole-cell protein extracts were prepared with the Active Motif Nuclear Extract Kit (Active Motif Europe) according to the manufacturer’s protocol. Protein concentrations were determined with the BCA (bicinchoninic acid) protein assay kit (Pierce, Thermo Scientific) with bovine serum albumin (BSA; Pierce) as the standard control. For Western blots, 40 µg of protein for each sample was separated by electrophoresis on 10% sodium dodecyl sulfate polyacrylamide gels (Invitrogen Corp.) with transfer to nitrocellulose membranes using the iBlot™ DryBlotting device (Invitrogen Corp.) and iBlot™ Transfer stacks. Antibody labeling was detected using relevant secondary antibodies conjugated to horseradish peroxidase (HRP) (1:4,000; DAKO Cytomation) and enhanced chemi-luminescence solutions (GE Healthcare ECL/ECL Plus (Amersham)). For quantitative analysis, the bands were scanned in a UVP GelDoc-It imaging system, and the band densities measured with Labworks 4.6 software (Bio-Rad Lab.).

### Global gene expression profiling

RNA quality was determined using the RNA 6000 Nano Assay^TM^ kit with Agilent 2100 BioanalyserTM and 2100 Expert Software (Agilent Technologies). RNAs with a RNA integrity number (RIN) score of ≥ 9 were converted to single stranded cDNA, using the Ambion^TM^ WT Expression Kit (Ambion) and then fragmented and labelled using the Affymetrix GeneChip^TM^ WT Terminal Labeling Kit (Affymetriz). Samples were hybridized to Affymetrix^TM^ Human Gene 1.1 ST Arrays using the GeneTitan^TM^ Instrument following the manufacturer’s instructions.

Data was processed with the Robust Multichip Analysis (RMA) algorithm [16], as implemented in Affymetrix Power Tools (APT, version 1.15.0), to generate normalized transcript cluster signal values. Systematic variation in expression between time points and conditions was modelled using LIMMA [17]. Three contrast matrices were generated: extracting contrasts between time-points under control conditions; extracting contrasts between time-points under knockdown conditions; and identifying transcript clusters that respond differently over time between the two conditions. *P*-values were adjusted using Benjamini and Hochberg’s method to control the false discovery rate (FDR) [18]. Universally low expressed transcript clusters (below the median across all 48 arrays) were excluded.

Functional annotation and enrichment testing was performed using the DAVID Gene Functional Classification Tool, version 6.7 [19, 20]. Unique Entrez IDs represented on the HuGene 1.1 ST array were employed as a background, excluding universally low expressed transcript clusters. *P*-values were adjusted using Benjamini and Hochberg’s method [18].

### Investigation of tool MAP3K8 inhibitors

A549 cells were pre-treated with putative MAP3K8 inhibitors for one hour before IL-1β stimulation (1ng/ml) for 24 h. Cytokine measurements were made in supernatants as detailed above. Cells for proteins extraction and Western blots were harvested after 30 min IL-1β stimulation.

### Statistical analyses

Statistical comparisons between treatments were carried out using Mann-Whitney U tests for nonparametric data, comparing medians of samples to controls. Means were compared by Student’s t test. Comparisons between more than two groups were carried out using Kruskall-Wallis (ANOVA) tests. A *P* value of < 0.05 was considered significant. All statistical analyses were performed using the Statistical Program for Social Sciences (SPSS 17.0 for windows, SPSS Inc., Chicago, IL, USA) and graphs were drawn using GraphPad Prism Version 5.01 software (GraphPad Software Inc, California, USA).

## Results

### MAP3K8 activation and silencing in A549 cells modulates IL-1β-induced cytokine expression

**Fig. 1:**
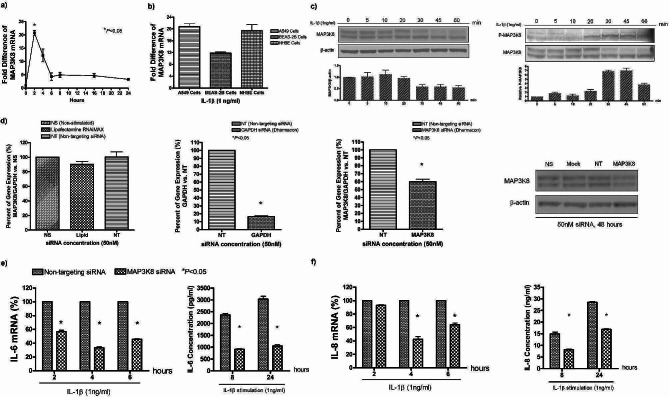
MAP3K8 activation and silencing in A549 cells modulate IL-1β-induced cytokine expression. **a)**1 ng/ml of IL-1β induced MAP3K8/GAPDH mRNA expression. in A549 cells, **b)***MAP3K8* gene expression in A549, BEAS-2B and NHBE cells at 2 h after IL-1β stimulation, **c)** MAP3K8 phosphorylation (P-MAP3K8) protein expression induced by IL-1β in A549 cells, **d)** siRNA knockdown of *MAP3K8*, including reagent control, GAPDH positive control, *MAP3K8* gene and protein knockdown efficiency at 2 h and 48 h, respectively **e**,** f)***MAP3K8* knockdown reduces IL-6 and IL-8 mRNA expression at 2, 4 and 6 h, and their protein expression at 8 and 24 h

Firstly, we examined *MAP3K8* transcript levels after IL-1β stimulation by real-time PCR in A549 cells. This showed a 20-fold (4.2 log2) rise in MAP3K8 that peaked 2 h after stimulation (Fig. 1a), confirming our previous global gene profiling in the cells. We found a similar rise in *MAP3K8* gene expression in NHBE cells and in BEAS-2B cells (Fig. 1b). We analysed IL-1β-induced activation of MAP3K8 in A549 cells by Western blotting, showing MAP3K8 to be degraded at 30 min and phosphorylation of MAP3K8 to occur at 20 min after stimulation (Fig. 1c). These results are consistent with previous observations in knockout mice [21].

After optimization we obtained partial silencing of *MAP3K8* in A549 cells by siRNA (Fig. 1d). Silencing of *MAP3K8* resulted in a significant reduction in mRNA expression and protein release of IL-6 and IL-8 (Fig. 1e and f) after IL-1β stimulation. The reduction was not present in controls transfected with non-targeted siRNA.

### MAP3K8 regulates ERK, SAPK/JNK pathways but not the p38 MARK pathway

**Fig. 2:**
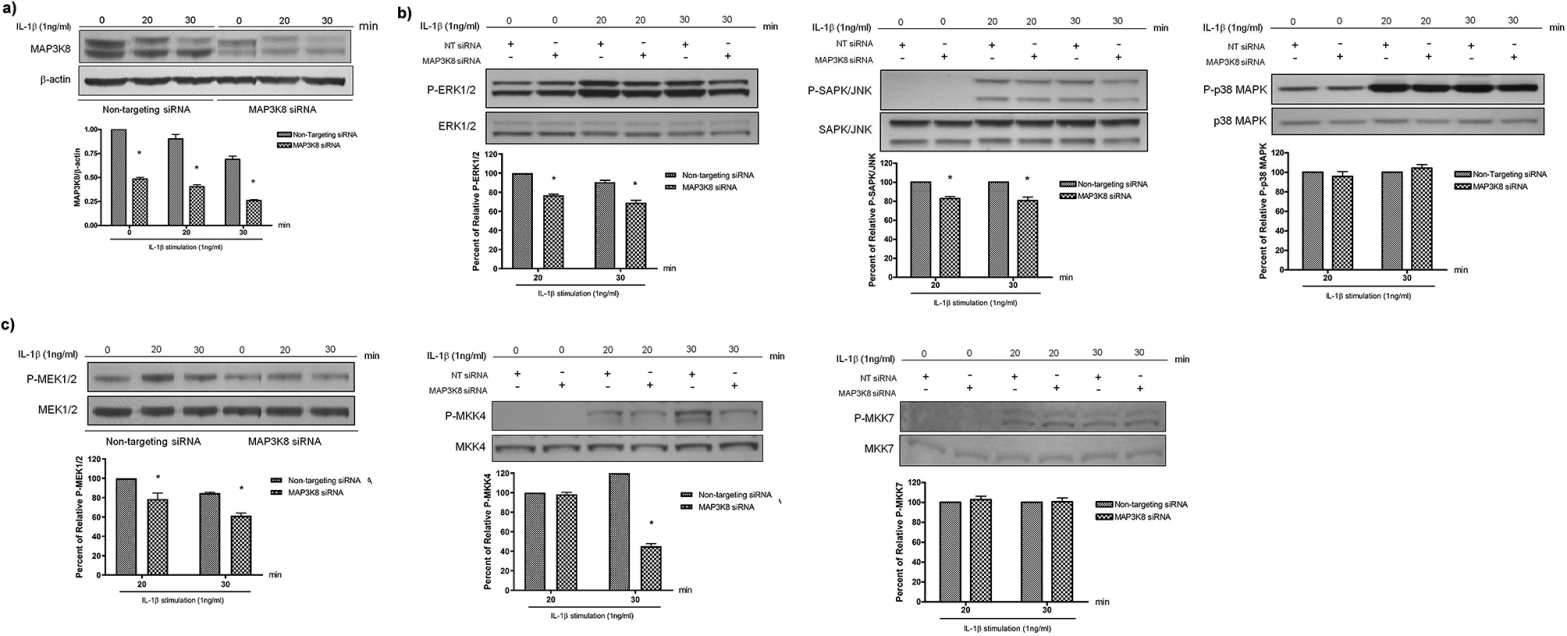
MAP3K8 regulates MAPK signalling pathways of ERK/MAPK and SAPK/JNK but not the p38 MAPK. **a)**RNAi-mediated knockdown of *MAP3K8* results in consistent reduction of MAP3K8 protein expression in the presence of 1 ng/ml IL-1β, assessed at 20 and 30 min after IL-1β stimulation, **b)** IL-1β-activated MAP3K8 regulates the phosphorylation of ERK1/2 and SAPK/JNK, but not p38 at 20 and 30 min after IL-1β stimulation, **c)** IL-1β-activated MAP3K8 regulates the phosphorylation of MEK1/2 and MKK4 but not MKK7 in A549 cells, MAPK phosphorylation was assessed and compared between non-targeting siRNA and *MAP3K8* siRNA group. Data are expressed as the mean ± SEM of at least three separate experiments. The gels show repeat labelling of Western blots with different antibodies: each section represents an individual blot, with each antibody stain in a separate box

We examined downstream kinases of MAP3K8 with wild-type and silenced A549 cells following IL-1β stimulation. MAP3K8 expression was significantly decreased within 30 min in silenced cells (Fig. 2a) We observed an approximately 30% decrease in phosphorylation of ERK1/2 (P-ERK1/2) and a 20% decrease in phosphorylation of SAPK/JNK (P-SAPK/JNK) at 30 min in after IL-1β stimulation in the presence of *MAP3K8* knockdown but there was no detectable difference in P-p38 MAPK between *MAP3K8* gene silencing and controls (Fig. 2b). We observed similar patterns in MEK1/2 and MKK4, in the intermediate pathways between MAP3K8 and of ERK1/2 and SAPK/JNK respectively, but no difference in phosphorylation of P-MKK7 in the SAPK/JNK pathway was observed (Fig. 2c).

### *MAP3K8* knockdown enhances dexamethasone suppression of IL-1β-induced cytokine production in A549 cells

Our observation that human MAP3K8 activation has minor effects on MKK7 and p38 MAPK phosphorylation supports the suggestion that MAP3K8 may be a better anti-inflammatory target than the extensively investigated p38α MAPK [22].

**Fig. 3:**
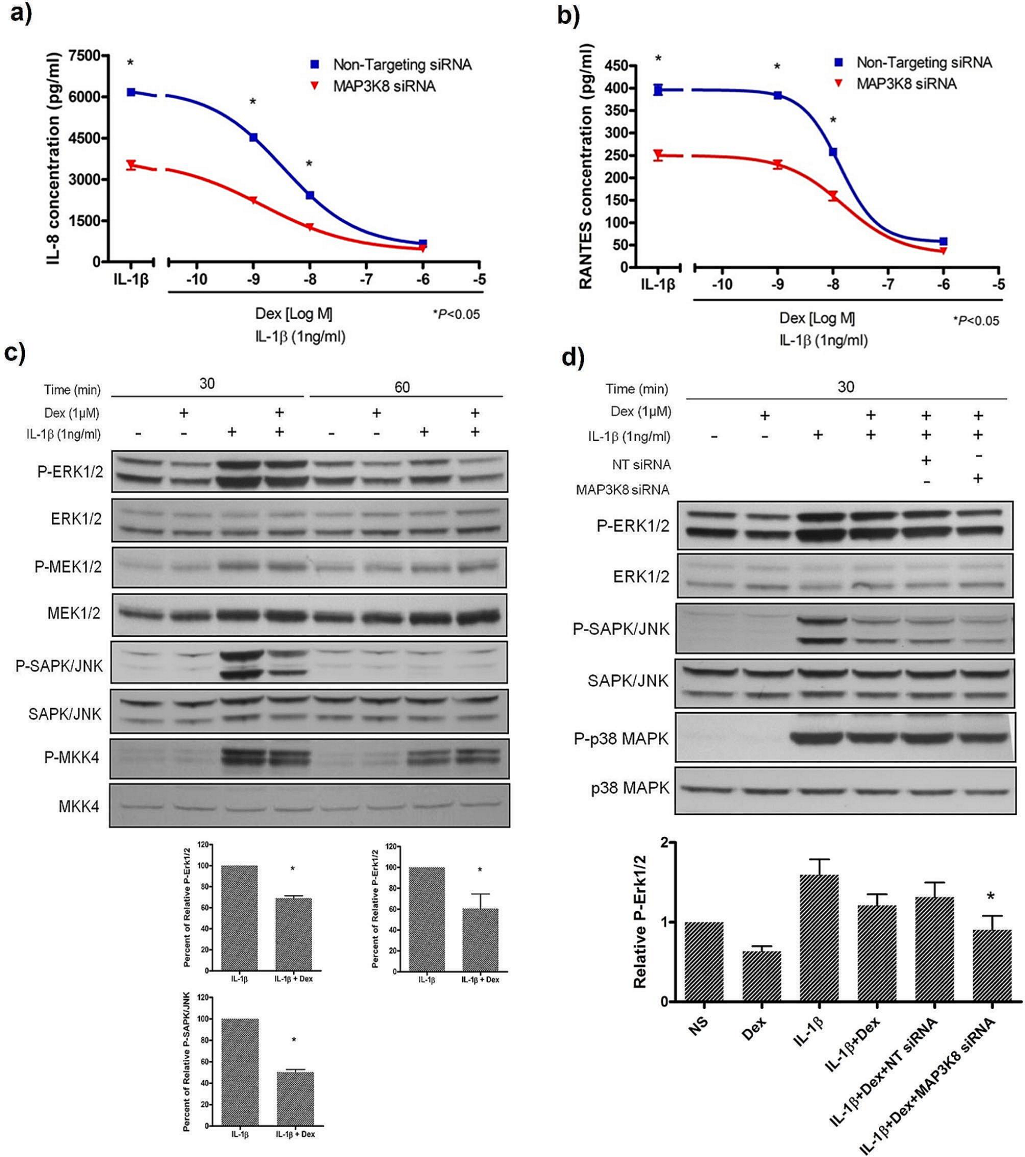
*MAP3K8* knockdown enhances dexamethasone suppression of IL-1β-induced cytokine production in A549 cells. **a)***MAP3K8* knockdown reduces IL-8 and **b)** RANTES supernatant release from A549 cells pre-treated with dexamethasone at 24 h after 1 ng/ml IL-1β stimulation, **c)** Dexamethasone effects on phosphorylation of ERK/MAPK and SAPK/JNK but not their upstream MEK1/2 or MKK4 analysed by Western blotting at 30 min after IL-1β stimulation, **d) ***MAP3K8* knockdown enhances the inhibition of phosphorylated ERK1/2 and SAPK/JNK but not p38 MAPK at 30 min after IL-1β stimulation in the presence of dexamethasone (µM) pre-treatment. (All mean ± SEM of at least three separate experiments). The gels show repeat labelling of Western blots with different antibodies

Glucocorticoids are the mainstay of therapy for inflammatory diseases, and so we measured the dose-response curve of dexamethasone suppression of IL-1β-induced IL-8 and RANTES production (Fig. 3a and b) in our A549 model. We observed that the curve was moved one log10 downwards in the *MAP3K8* siRNA group, indicating MAP3K8 to act through dexamethasone-independent mechanisms.

We also found that dexamethasone reduced the appearance of phosphorylated ERK1/2 and SAPK/JNK, but not their upstream kinases MEK1/2 and MKK4 (Fig. 3c). The combination of dexamethasone and *MAP3K8 *gene silencing resulted in a greater inhibition of phosphorylated ERK1/2 and SAPK/JNK, but not p38 MAPK (Fig. 3d). These results suggested that *MAP3K8* knockdown enhanced the effect of dexamethasone through inhibition of MAPK upstream kinases.

### Time series analysis of gene expression reveals effects of *MAP3K8* knockdown in IL-1β-stimulated A549 cells

We next performed a time series analysis to profile the global gene expression response to partial *MAP3K8* knockdown versus non-targeting siRNA controls in the A549-IL-1β model. We compared the expression changes for two groups at 0, 1, 2, 4, 6, 8, and 12 h, and a total of 46 genes showed significantly different response (Table 1, P corrected < 0.05). Nineteen genes (*P* corrected < 0.01) demonstrated a distinct altered temporal response to IL-1β stimulation following suppression of MAP3K8. The matrix metalloproteinases *MMP1*, *MMP3* and *MMP10* showed a blunted response to IL-1β following knockdown, as did other components of the extracellular matrix such as *ITGB8*,* LAMC2*, and *PLAT*.

**Table 1:** Genes altered by siRNA knockdown of *MAP3K8* in A549 cells after IL-1β stimulation of A549 cells

ID	Dif1	Dif2	Dif4	Dif6	Dif8	Dif12	Ave Expr	adj. *P*.Val	Gene Assignment	Function Group	
8,149,825	-0.0297	-0.5986	-0.7126	0.2788	0.3121	0.2948	9.7303	7.78E-12	*STC1*	Growth factors	
7,926,900	-0.0312	-0.6082	-0.1792	0.1979	0.0593	0.2146	9.1270	2.17E-06	*MAP3K8*	ERK pathways	
8,139,207	-0.3252	-0.6912	-0.3870	0.0652	0.0955	0.3487	6.6862	1.72E-05	*INHBA*	Growth factors	
8,029,693	-0.5578	-1.3920	1.2786	0.3235	0.1937	-0.2268	6.5645	1.88E-05	*FOSB*	ERK pathways	
7,951,284	0.2147	-0.2445	-0.9981	-0.0006	-0.0489	0.8034	4.4087	0.00010247	*MMP3*	Extracellular proteases	
8,150,509	0.2576	-0.4648	0.1007	0.2679	0.1877	0.2294	9.0473	0.0002872	*PLAT*	Extracellular proteases	
7,951,259	0.5534	-0.9547	-0.5190	0.2045	-0.3736	0.3734	6.3240	0.00045375	*MMP10*	Extracellular proteases	
8,153,201	0.0479	-0.4685	-0.1152	-0.0437	0.3042	-0.2654	10.4016	0.00045375	*EIF2C2*		
7,917,649	0.2127	0.0377	0.1270	-0.3302	-0.2098	-0.1283	8.7129	0.00093737	*TGFBR3*	Growth factors	
7,944,769	-0.0572	-0.0887	-0.2799	-0.0066	0.3259	0.1219	10.4395	0.0009716	*GRAMD1B*		
8,095,728	0.1828	-0.2296	-0.2194	-0.1545	0.0599	0.3256	11.6033	0.00175164	*EREG*	Growth factors	
7,996,772	-0.0158	0.0041	-0.0028	-0.2563	-0.0490	-0.0799	9.8132	0.00259827	*SLC7A6*		
8,154,245	-0.4487	0.2877	0.0338	1.1178	-0.4189	-0.2688	5.9701	0.00317684	*PDCD1LG2*	ERK pathways	
8,124,166	0.1576	-0.0397	0.2982	0.2472	-0.2132	0.0147	7.8654	0.005414	*MBOAT1*	Lysophopholipids	
7,957,298	-0.1566	-0.0458	-0.3356	0.0307	0.2926	0.0490	9.7235	0.00550053	*NAV3*		
7,951,271	-0.3148	-0.4711	-0.5804	0.1105	-0.1583	0.5121	7.2633	0.00550053	*MMP1*	Extracellular proteases	
8,091,600	0.1084	-0.0836	0.1036	0.4321	-0.1186	-0.0600	7.4795	0.00550053	*PLCH1*	Lysophopholipids	
7,902,227	0.0436	0.2069	0.3442	0.0419	-0.5557	-0.0956	7.0090	0.00998721	*GADD45A*	ERK pathways	
8,057,677	0.0052	0.1962	-0.0289	-0.4795	0.0687	-0.0085	8.1113	0.00998721	*SLC40A1*	Iron metabolism	
7,997,401	0.0344	-0.1522	0.1799	0.2824	0.2857	-0.0481	7.6882	0.01063101	*BCMO1*		
8,054,722	0.2205	-0.9733	-0.1205	-0.0332	0.2151	0.5247	6.2915	0.01063101	*IL1B*		
8,152,617	0.0279	-0.1290	-0.3494	-0.2234	0.2312	0.2599	8.8467	0.01211707	*HAS2*	Extracellular matrix	
8,123,609	0.0341	-0.5227	-0.0309	0.1276	0.0553	0.1139	10.1999	0.01306179	*SERPINB9*	Extracellular matrix	
7,961,371	0.0566	-0.2009	-0.1672	-0.0378	-0.0165	0.0102	9.8130	0.01434946	*DUSP16*	ERK pathways	
7,909,214	0.1965	-0.1592	0.0568	0.6090	0.1808	-0.4726	7.2441	0.01434946	*RASSF5*	ERK pathways	
7,908,072	0.1938	-0.2754	-0.2798	0.1108	-0.0594	0.1371	9.7632	0.01434946	*LAMC2*	Extracellular matrix	
8,131,666	-0.1251	-0.4561	0.0721	-0.0613	-0.0307	-0.0677	8.2473	0.01434946	*ITGB8*	Extracellular matrix	
8,146,579	0.0335	0.0855	-0.0022	-0.2335	-0.1355	-0.0524	7.9175	0.01434946	*CHD7*		
8,162,940	-0.1683	0.1241	-0.0298	-0.1565	-0.0990	-0.2986	8.7714	0.01759226	*ABCA1*		
8,160,521	0.0144	0.0895	0.1278	0.3431	-0.2911	-0.1801	8.5845	0.01906981	*MOB3B*		
8,021,635	0.1217	0.0319	-0.4090	-0.3232	0.3902	0.1589	3.6854	0.02025975	*SERPINB2*	Extracellular matrix	
7,987,454	0.1111	0.0995	0.3137	-0.5803	-0.1220	-0.1906	7.2998	0.02025975	*BMF*		
8,040,365	0.0105	0.0497	-0.2679	0.0267	-0.7976	0.7653	4.1035	0.0209142	*TRIB2*		
7,951,309	-0.0491	-0.1362	-0.6825	0.1977	0.0703	0.2436	3.9447	0.02471666	*MMP13*	Extracellular proteases	
8,072,678	0.1496	-0.0771	-0.3122	0.0068	0.5784	-0.1840	9.9875	0.02655837	*HMOX1*	Iron metabolism	
8,073,214	-0.0930	0.0348	-0.0701	-0.2201	0.0692	-0.2012	8.1931	0.02655837	*TNRC6B*		
7,909,332	-0.1267	0.0167	-0.1388	-0.2643	-0.0820	0.4067	10.1092	0.02655837	*CD55*		
7,956,009	-0.0204	0.0449	-0.1819	0.0758	0.4650	0.1654	8.1114	0.02655837	*METTL7B*		
7,917,347	0.0433	-0.0119	0.2583	-0.1106	-0.2745	-0.2573	9.1879	0.03022086	*DDAH1*		
8,132,694	-0.1352	0.0592	-0.1252	-0.3574	0.2755	0.2553	9.9915	0.03722356	*IGFBP1*	Growth factors	
7,954,077	0.0697	0.0799	-0.0287	-0.1959	-0.2314	-0.1436	8.0405	0.03868964	*KIAA1467*		
8,121,257	0.0055	-0.3574	-0.0367	0.2346	-0.0094	-0.3641	8.7712	0.04079342	*PRDM1*		
8,007,931	-0.0643	-0.0290	0.0077	-0.3214	-0.1882	0.0141	6.1123	0.04321407	*ITGB3*		
7,946,478	-0.0081	-0.0778	-0.1677	-0.1382	0.4313	-0.2799	9.9379	0.04328626	*DENND5A*		
7,922,229	-0.1620	-0.7991	-0.0055	0.6619	0.2436	0.0700	5.4737	0.04535622	*SELE*		
8,129,861	-0.0997	-0.1270	-0.1209	-0.2175	0.0590	-0.0593	9.2467	0.04811681	*IFNGR1*		

Ave. Expr: average expression; adj.P. Val: adjust *P* value. Dif1 – Dif12 are the log2 fold changes associated with each contrast. The contrasts were specified as follows (where Knockdown/Control are the conditions, and 0–12 are the time points of the hours after stimulation):

Dif1=(Knockdown.1 h-Knockdown.0 h)-(Control.1 h-Control.0 h);

Dif2=(Knockdown.2 h-Knockdown.1 h)-(Control.2 h-Control.1 h);

Dif4=(Knockdown.4 h-Knockdown.2 h)-(Control.4 h-Control.2 h);

Dif6=(Knockdown.6 h-Knockdown.4 h)-(Control.6 h-Control.4 h);

Dif8=(Knockdown.8 h-Knockdown.6 h)-(Control.8 h-Control.6 h);

Dif12=(Knockdown.12 h-Knockdown.8 h)-(Control.12 h-Control.8 h).

### Effectiveness of MAP3K8 inhibitors in A549 cells for inflammatory cytokine suppression

**Fig. 4:**
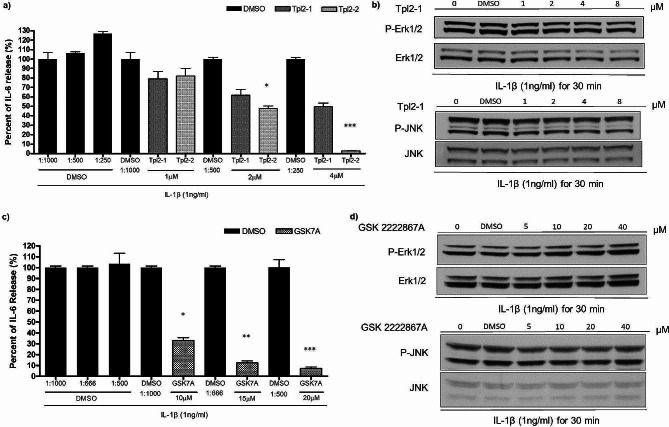
Investigation of MAP3K8 inhibitors. **(a)** A549 cells pre-treated with Tpl2 inhibitors for one hour and cells were stimulated with IL-1β (1ng/ml) for 24 h. Results are shown for IL-6 production (similar results for IL-8 not shown). **(b)** A549 cells pre-treated with MAP3K8 inhibitors for one hour and proteins for Western blot collected after IL-1β stimulation for 30 min. P-ERK and P-JNK signalling appear unaffected. **(c)** A549 cells pre-treated with GSK2222867A for one hour and stimulated with IL-1β (1ng/ml) for 8 h. Results are shown for IL-6 production (similar results for IL8 not shown). The compound GSK2222284A produced no change in cytokine production in the same doses (data not shown). (d) A549 cells pre-treated with GSK2222867A for one hour and proteins for Western blotting collected after IL-1β stimulation for 30 min. P-ERK and P-JNK signalling appear unaffected. The gels show repeat labelling of western blots with different antibodies: sections **b)** and **d)** each represent an individual blot, with each antibody stain in a separate box

We assayed the effects of publicly available putative MAP3K8 inhibitors including Tpl2-1, Tpl2-2, and GSK2222867A. Tpl2-1 is a reversible competitive inhibitor of MAP3K8, TpL2-2 is a potent ATP-competitive inhibitor of MAP3K8. GSK2222867A is specifically inhibits the ability of MEK to phosphorylate ERK without affecting the JNK and p38 pathways. These compounds only showed inhibition of IL-6 production at high doses (1–10 µmol) (Fig. 4a and c, similar results of IL-8 not shown). In contrast to *MAP3K8* siRNA knockdown, we did not observe inhibition of ERK and JNK phosphorylation (Fig. 4b and d). These findings suggest a need for further development of MAP3K8 inhibitors.

## Discussion

MAP3K8 is revealed to be a hub in the initial inflammatory response in human epithelial cells. MAP3K8 forms a ternary complex with NFKB1/p105 and TNIP2. It interacts with NFKB1; the interaction increases the stability of MAP3K8 but inhibits its MEK phosphorylation activity, whereas loss of interaction following lipopolysaccharide (LPS) stimulation leads to its degradation [21]. MAP3K8 also interacts with CD40 and TRAF6; the interaction is required for ERK activation and interacts with KSR2; the interaction inhibits ERK and NF-kappa-B activation [23–25]. Although the MAP3K8 signalling pathway has been well documented, it is still of interest to know to how the molecule regulates inflammation in airway epithelium and what other molecules might be involved in its pathway.

In this report, we confirm that knockdown of *MAP3K8* can significantly reduce release of the inflammatory cytokines IL-6 and IL-8 after IL-1β stimulation. It reduces the phosphorylation of ERK1/2 (P-ERK1/2) and phosphorylation of SAPK/JNK (P-SAPK/JNK) but not p38. We observed similar patterns in MEK1/2 and MKK4, in the intermediate pathways between MAP3K8 and of ERK1/2 and SAPK/JNK respectively, but there was no difference in phosphorylation of P-MKK7 in the SAPK/JNK pathway.

Asthmatic patients can show early and late immune response to allergens and other stimuli. Our finding show that human epithelium responds quickly through the MAP3K8 pathway and that downstream signals are through MEK/JNK but not p38. Although many investigations have found p38 MAPK activities in blood and in airway epithelial cells to be increased in severe asthma [26, 27], in our data p38 did not respond in the early hours of IL-1β stimulation, suggesting that it is not a major pathway for cytokines stress.

Our study also provides novel insights from global gene expression profiling. We have shown that knockdown of *MAP3K8* dramatically influences the expression of genes categorised as extracellular proteases, extracellular matrix, growth factors, ERK pathways, lysophopholipids and iron metabolism. The matrix metalloproteinases *MMP1*, *MMP3*, and *MMP10* showed a blunted response to IL-1β following knockdown, as did other components of the extracellular matrix such as *ITGB8*,* LAMC2*, and *PLAT*. MMPs are involved in wound healing, inflammatory cell trafficking and tissue remodelling and repair [28], and their down-regulation may be of therapeutic value [29].

The largest up-regulated difference was in *PDCD1LG2*, an immune inhibitory molecule expressed on activated T-cells, suggesting MAP3K8 inhibition may have the potential to inhibit T cell cytokine production [30].

Our results are consistent with previous studies in mice, where *Map3k8* (Tpl2) ablation revealed an important role in ERK signalling [5, 31, 32] and shown MAP3K8 to be a strong regulator of pro-inflammatory function [33, 34] and the expression of Th2 cytokines [35].

Our investigations had the following advantages: Firstly, they showed that inhibiting MAP3K8 can directly reduce inflammatory cytokine release and can enhance the anti-inflammatory effects of dexamethasone. Secondly, we showed that MAP3K8 directly influences phosphorated MEK and JNK pathways but is distinct from p38 MAP pathway. We also showed knockdown of *MAP3K8* can influence extracellular proteases, extracellular matrix, growth factors, ERK pathways, lysophopholipids and iron metabolism.

Despite a wealth of positive results supporting the role of MAP3K8 in airway inflammation, the putative MAP3K8 inhibitors including Tpl2-1, Tpl2-2, and GSK2222867A showed only partial inhibition of inflammatory responses in our models, suggesting further screening of small molecules for inhibition of MAP3K8 is necessary.

## Conclusions

This study captures the dynamic temporal effects of MAP3K8 that underly inflammation in epithelial cells. MAP3K8 regulates MEK and JNK pathways and can regulate expressions of important matrix metalloproteinases in the epithelium. Inhibition of MAP3K8 can significantly reduce inflammatory responses in epithelial cells and can enhance the anti-inflammatory effects of the steroid dexamethasone. Although the results presented here were obtained in an airway epithelial cell model, the results may provide many potentially druggable targets for other epithelial inflammatory diseases.

## Data Availability

The transcriptomics dataset is available in the NCBI database with accession number GSE103987.
